# The vulnerable and the susceptible: The weight of *evidenza* to stop exploiting activities generating toxic exposures in unprotected and deprived countries

**DOI:** 10.7189/jogh.11.03046

**Published:** 2021-03-27

**Authors:** Chiara Frazzoli

**Affiliations:** Department of Cardiovascular and Endocrine-Metabolic Diseases, and Ageing, Istituto Superiore di Sanità, Rome, Italy

## THE VULNERABLE AND THE SUSCEPTIBLE

Healthy people who practice primary prevention of diseases in different economic, social, environmental contexts have different chance of health due to different determinants of health [1]. Primary prevention focuses on individual risk factors (behavioral risk factors like eg, smoking, consuming alcohol, inappropriate use of hazardous products; genetics) and proactivity in non-clinical life choices like the health pyramid (diet, physical exercise, meditation). The ethiology of diseases focuses on both primordial and primary prevention. Primary prevention aims at managing specific risk factors and improving protective factors to reduce the incidence of diseases; while primordial (or primal) prevention aims at establishing and maintaining conditions that prevents such risk factors.

We could state that primary prevention points at protecting biological vulnerability (that equally characterizes all beings, including particularly vulnerable life stages, eg, *in utero* life and early infancy), while equal determinants of health should be guaranteed by primordial prevention.

Primordial prevention is very dependent on the commitment and determination of individual government. Prioritising prevention is a matter of political and cultural choices, but also of resources. A main constraint for prevention in low-income countries is the limited recognition and availability of scientific evidence. In general, countries that are poor in scientific evidence are “unprotected” by public health policies [2]; countries without resources for scientific research are generally those “deprived” based on the dimensions of poverty (health, education, standard of living) and relevant indicators (nutrition, child mortality, years of schooling, school attendance, cooking fuel, sanitation, drinking water, electricity, housing, information and transport) [3]. Without public health policies assuring primordial prevention, people are not only biologically vulnerable but also susceptible. This means that their weakness towards hazards is higher than the biological vulnerability of those living in areas protected by primordial prevention. For instance, malnutrition in deprived populations makes people susceptible, not only vulnerable, to the effects of toxic substances. In its turn, toxic exposures in unprotected countries aggravates the deficiency of essential and protective nutrients [4], increases disabilities, non-communicable diseases, communicable diseases (eg, AIDS) and failure of treatments.

As stated by Michael Kottow [5], by mislabelling susceptibility with biological vulnerability (that recalls the difference between equity and equality) the global ethical obligation to guarantee equity in protecting chance of healthy life for all becomes less evident and obvious. While socio-economically developed countries increase their safety standards (eg, [6]), the maintenance of their high consumption rates (without reducing waste and changing habits) in all sectors (food, non-food) means exportation or dumping of hazardous materials (eg, wastes of technological devices; consumer products that have been banned for safety reasons; agrozootechnical products) or exploitation of intensive activities (eg, mining, agriculture for import purposes) in deprived countries where prevention is insufficiently structured to protect people. Exploitation of these activities is made possible by unequal health protection policies and goes on despite whole population's and their progeny's high susceptibility to health damage [7].

## EVIDENCE-BASED PREVENTION AND *EVIDENZA*

Evidence-based prevention, ie, the process of making decisions based on the best available scientific evidences, should not lend itself as a tool for unethical actors to exploit the lack of scientific evidence in countries where scientific research is still obstructed by costs and infrastructures. This is particularly true for toxic exposure assessment, which is usually difficult and costly.

In science, “evidence” is the sum of scientific observations corroborating or rejecting a hypothesis. Lack of local data/scientific evidence (and therefore of evidence-based prevention) cannot be the reason for actions that are prohibited and unethical in high-income (and scientifically advanced) countries. In most cases, it is not necessary to repeat hazard characterization: up-to-date standards developed by international agencies are universally applicable. For instance, this approach is successfully adopted in highlighting the market pressure and unethical dumping of baby bottles that are banned in economically advanced countries and exported in emerging markets where consumers are in need of all [8]. Lots of new products are progressively substituting the local artisanal goods, eg, clothes made from artificial fibres treated with chemical dyes and flame retardants, phthalates-containing PVC toys, detergents and cosmetics, perfluorinated-containing stain-resistant carpets, medical devices, catalytic vehicles in areas known to fuel vehicles with lead-based petrol [9]. Moreover, benchmarks that are protective in economically advanced countries could not be protective in living scenarios that are more stressed by poverty, and therefore should be lowered. The epidemiologic approach “further studies are necessary” should be replaced therefore by “although the evidence is incomplete, it is strong enough to recommend risk prevention measures, especially in deprived contexts and among malnourished people”. Toxic waste like e-waste (electric and electronic waste) is dumped because of the lack of local scientific evidence and protection by national public health. It causes widespread and severe contamination of all environmental compartments, with long lasting dramatic consequences [10]. E-waste entrains exposure to mixtures of endocrine disrupters, neurotoxicants, and immunotoxicants (eg, chemical elements, nanoparticles, polycyclic aromatic hydrocarbons, brominated flame retardants, dioxin-like and non-dioxin-like polychlorinated biphenyls, polychlorinated dibenzo-p-dioxins and polychlorinated dibenzofurans) impairing health of the present and future generations [7]. In general, in breastfed infants who live in communities where they have chronic contact with e-waste, exposure to the most potent groups of dioxin-like chemicals is dozens of times higher than WHO limits [7]. Even in the absence of complete (and costly) environmental and human internal exposure data, environmental fate of toxicants is known and predictable. The same applies to pesticides and other products used to boost agricultural productivity: their unrestrained use for both domestic and export purposes will easily lead to unsafe human and environmental exposures [4].

**Figure:**
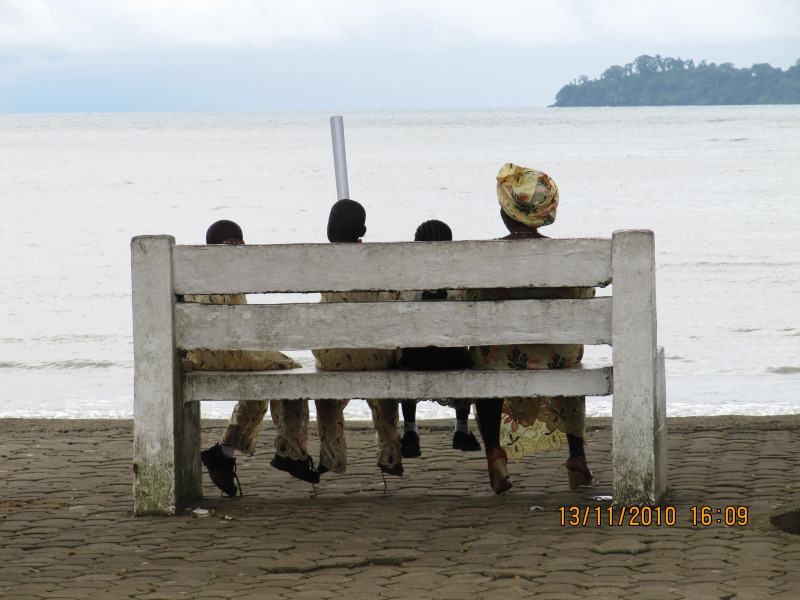
Photo: Risks for health in low-income countries are exacerbated by deprivation (including malnutrition) and lack of primordial and primary prevention. Limbe, Cameroon, 2010. (from the author's own collection, used with permission).

As argued by Franco Berrino [11], the Italian language offers the word “evidenza” that means something different from the English word “evidence”. *Evidenza* means *the quality of everything that you understand immediately without needing proof*. Based on scientific knowledge of toxicological risk factors, direct observation of materials, products, behaviour and practices eg, during production, transport, handling, use/consumption, and disposal is a low cost approach that makes formal risk assessment less needed in many situations [9]. *Evidenza* can feed the international ethical management of risk factors to protect the chance of health for all, considering both vulnerability and susceptibility.

## EMERGENCY TODAY FOR SEVERE HEALTH OUTCOMES TOMORROW

*Evidenza* shows even more than this. The deprived life environment of infants and children makes these life stages (particularly vulnerable to toxic effects due to the development phase of nervous, immune and endocrine systems, and of organs and tissues that are also less able to detoxify) multi-factorially and chronically exposed to severely toxic mixtures at higher levels than adults (more food, water, air per body weight) [12]. In the current UN International Year for the Elimination of Child Labor, the emergency nature of toxic exposures (eg, in mining, e-waste recycling and disposal, but also in intensive agriculture) should be stated. Often without adequate protection, child labor (but also child and infant living) in these and other highly severely toxic environments (eg, gas flaring in crude oil exploitation) makes this acute and chronic high exposure an emergency issue. Not readily discernible today in their health impacts (long-term, transgenerational), the emergency nature of these exposures is particularly inherent to the short life-stage windows (pre-, peri- and early post-natal life, childhood) associated to high risk of compromised healthy adulthood (cancer and other non-communicable diseases) and communicable diseases [4,7,12]. These emergencies overwhelm local risk management capacity in terms of characterization of safe areas for agriculture and living [13,14], and costly long-range environmental remediation. The persistence of unethical activities that continue to accumulate pollutions complicates the task even more. Most toxicants are persistent (with consequent additive or synergistic effects of mixtures) and bio-accumulate in living organisms; they also bio-magnify in food chains [15]. They are transferred from soils to plants to people, from soils to plants to animals (eg, through carry-over in grazing food producing animals) to people, or even directly from soils to people in geophagic communities [16]. In general, acute and chronic severe environmental pollution involve people occupationally exposed, people living in the area, but also the population at large: in fact, the ecosystem makes the contamination of food producing animals and water a source of daily exposure for the general population [15]. In heavily polluted areas, exposure exceeds the detoxification rate and continuously increases the body (and environmental) burden; malnutrition exacerbates the scenario [4]. An impressive example is the difficulty in finding Lead “unexposed” control subjects, Lead was even found in children, for biomonitoring studies in Nigeria [17]. Such kind of “congenital body burden” of neurotoxicants also aggravates stigma in some circumstance, thus feeding the vicious circle of destitution. The absence of informed choice, awareness of healthy alternatives (when easily available) and good practices in handling, use or recycle hazardous materials in contact with food and at the environment-food chain interface is a poverty-related susceptibility factor. The global market should not exacerbate the exposure scenario with consumer products banned elsewhere for safety reasons. Indeed, without immediate international responsibility and action, both environmental emergencies and unsafe market products in the emerging African market are perpetuating the cycle of poverty and poor chance of health of large numbers of people.

## PRIMORDIAL AND PRIMARY PREVENTION

Determinants of heath, or structural factors of primordial prevention, are general socio-economic, cultural and environmental conditions covered by Sustainable Development Goals: from equality, justice, poverty, education and employment, to food security and nutrition (sustainable agriculture and food production), water and sanitation, health care services, housing, safe work environments, ecosystems, peaceful and inclusive societies, accountable and inclusive institutions [18]. There is often *evidenza* for such factors. They subtend living and working conditions which may determine multiple and aggregate exposures that make people susceptible. *Evidenza* can boost context-situated risk analysis and prevention strategies, which could be facilitated by participant observation in field anthropological research [19]. Traditional cultures may be unavailable to accept radical changes “from outside”, however a lot of improvement can be mediated by local scientific community [8,19]. For instance, young women in childbearing age, women planning pregnancy and pregnant women may prevent the intake of toxicants through good practices in handling and cooking food [20]. Toddlers could be prevented from specific exposure routes like ingestion of soil, house dust, and water during crawling, playing and bathing [12]. Exposure during first life stage results from the mother’s body burden. In primiparas, especially with older age living in severely polluted areas, the expected high body burden could unfortunately result in the need of comparative risk-to-benefit assessment of their breast milk, the perfect food for the newborn; in terms of toxic pollution, milk of multiparous nannies or alternative milk from farm animals (eg, young multiparous ruminants like cows and goats, or donkeys) lived elsewhere could be preferable [7]. Further to anthropological approach, ecology is also crucial to deal with social change at the base of the web of interrelations between humans, animals and environmental health (One Health) [9]. Solutions come from understanding of traditional cultural, social, political and economic dynamics subtending agricultural features (eg, main farm animal species and products, as well as size, distribution and type of farming) and dietary habits [19]. From situated perspective, the protection from contamination of feeds, pastures, and watering sources of food producing animals is feasible. For instance, in One Health perspective, grazing livestock should be moved to pastures and watering sources at tolerable distance from polluted sites to avoid contaminant carry-over in meat and milk. Analogously, poultry should be kept on controlled agricultural land in order to reduce contaminant carry-over in meat and eggs. The hunting of wild animals, including fishes and birds (especially predators), should be limited to pollution free areas. Agricultural lands (crops, cereals, vegetables) and animal-rearing activities (farming, aquaculture) near or in downstream rivers are primary objective of environmental remediation campaigns together with waterways used for domestic uses (drinking, cooking and washing) and farm irrigation. Local solutions certainly need international responsibility in recognizing how susceptible communities already suffer of deprivation, destitution and noxious exposures that make them defenceless and more prone to diseases and disabilities than vulnerable peers that are protected by primordial prevention.

## CONCLUSION

As proven by the current SARS-CoV-2 pandemics, responsibility of the global society on prevention issues is feasible; and it should start from the protection of an equal and fair chance for a healthy adulthood for all, the vulnerable and the susceptible. Risk profiles drastically change with the environment (including food and consumer products) in countries of all levels of socio-economic development. Global Health should boost commitment and determination of governments in primordial and primary prevention. Unlike the developed countries, where risk transitions (eg, industrialization; globalization) occur in prospering economies and resources for science and public health, risk transitions in developing countries occur in settings of deprivation and lack of protection. Over-consumption, unsustainable exploitation of natural resources, and increasing waste production in high income countries strongly contribute to climate change and also to health disparities. The circular economy is an obligatory solution, provided that it is accompanied by the assessment of the impact on One Health. Like susceptible groups in high resource setting, developing countries deserve a science-based solidarity. In the current global reflection on guiding drivers (health, economy) and their balance for wholesome and sustainable development, concepts like primary vs primordial prevention, susceptibility vs biological vulnerability, equity vs equality, and scientific evidence vs science-based *evidenza* necessitate distinction. The increasing demand for safety should be balanced by reduced consumption, emissions and wastes in socio-economically developed countries. The relapsing on the health of other populations, especially children, of this unbalance must be made clear to the awareness of global society. The weight of *evidenza* requires to end the exploiting of activities generating toxic exposures in unprotected and deprived countries, to preserve the chance of health for all.
